# Post-settlement demographics of reef building corals suggest prolonged recruitment bottlenecks

**DOI:** 10.1007/s00442-022-05196-7

**Published:** 2022-06-04

**Authors:** Lauranne Sarribouette, Nicole E. Pedersen, Clinton B. Edwards, Stuart A. Sandin

**Affiliations:** Scripps Institution of Oceanography, UC San Diego, La Jolla, CA 92093-0202 USA

**Keywords:** Coral reefs, Demography, Palmyra atoll, Scleractinian, Structure-from-motion

## Abstract

For many organisms, early life stages experience significantly higher rates of mortality relative to adults. However, tracking early life stage individuals through time in natural settings is difficult, limiting our understanding of the duration of these ‘mortality bottlenecks’, and the time required for survivorship to match that of adults. Here, we track a cohort of juvenile corals (1–5 cm maximum diameter) from 12 taxa at a remote atoll in the Central Pacific from 2013 to 2017 and describe patterns of annual survivorship. Of the 537 juveniles initially detected, 219 (41%) were alive 4 years later, 163 (30%) died via complete loss of live tissue from the skeleton, and the remaining 155 (29%) died via dislodgement. The differing mortality patterns suggest that habitat characteristics, as well as species-specific features, may influence early life stage survival. Across most taxa, survival fit a logistic model, reaching > 90% annual survival within 4 years. These data suggest that mortality bottlenecks characteristic of ‘recruitment’ extend up to 5 years after individuals can be visually detected. Ultimately, replenishment of adult coral populations via sexual reproduction is needed to maintain both coral cover and genetic diversity. This study provides key insights into the dynamics and time scales that characterize these critical early life stages.

**Supplementary Information:**

The online version contains supplementary material available at 10.1007/s00442-022-05196-7.

## Introduction

Fluctuations in plant and animal populations through time result from differences in birth, growth, and mortality rates, and are described by an extensive body of theoretical and empirical work (Lande 1988; MacArthur and Wilson 1967; Pauly and Martosubroto 1980). Demographic rates tend to shift most dramatically as cohorts of individuals pass through the earliest life stages (Harper 1977; Hughes 1990; Silvertown and Charlesworth 2001; Vermeij and Sandin 2008). Thus, understanding the specifics of the timing of recruitment into adult populations is critical to the description of demographic fluctuations (Ricker 1954; Sandin and Pacala 2005). Quantitative estimates of the duration of the recruitment process are prerequisite as we consider pathways of population recovery following disturbances, approaches to maintain biological diversity, and strategies of wildlife management, among other topics (Clark et al. 1998; Holbrook et al. 2018; Hubbell et al. 1999).

For communities dominated by long-lived and slow-growing organisms, the study of recruitment dynamics is particularly timely as mortality events linked to climate driven disturbances are becoming increasingly severe (Anderson‐Teixeira et al. 2013; Hughes et al. 2019; Schweiger et al. 2020). Such systems typically possess an ensemble of strategies such as seed banks, clonality, and asexual propagation that allow rapid recovery following perturbation. However, centuries of local human pressures that are now exacerbated by global environmental changes have resulted in a lowered capacity for individuals to survive and contribute to ecosystem recovery (Adjeroud et al. 2017; Nolan et al. 2021; Stevens‐Rumann et al. 2018). As such, quantifying details of recruitment of sexually produced offspring into adult populations will become increasingly important for predicting potential demographic changes and guiding ecological communities toward configuration that are resilient to environmental change (Bernhardt and Leslie 2013; Thompson et al. 2009).

Recruitment is typically defined as the process of reaching the age beyond which individuals can be considered to have entered the adult population (Caley et al. 1996). For many organisms, recruitment is preceded by myriad post-settlement processes subjecting newly arrived individuals to a so-called “gauntlet” of challenges (Arnold et al. 2010). For sessile marine organisms in particular, the vulnerability of early life history individuals is particularly acute, as newly settled individuals experience disproportionately high rates of mortality relative to adults (Penin et al. 2010; Ritson-Williams et al. 2009; Vermeij and Sandin 2008). Survivorship of settling organisms is reduced dramatically through a number of impacts including smothering by overshading or sedimentation (Babcock and Mundy 1996; Maida et al. 1994), dislodgement on unstable substrate (Cameron et al. 2016; Chong-Seng et al. 2014), or competition with other sessile organisms (Vermeij and Sandin 2008). While it has been well noted that as sessile organisms grow and mature their susceptibility to such impacts lessens (Doropoulos et al. 2012; Raymundo and Maypa 2004), there are limited quantitative data estimating the duration of the functionally distinct ‘juvenile’ phase, i.e.*,* the period before recruitment.

Demographic studies conducted in marginal or degraded locations have indicated that current levels of recruitment will be insufficient to compensate for reductions in adult survivorship necessary to ensure population persistence for many groups of organisms (Bak and Meesters 1999; Chui and Ang Jr 2017; Guerrini et al. 2020; Hughes and Tanner 2000). Decline in adult survivorship can be particularly destabilizing when it occurs to structure-forming species such as reef-building corals which have experienced quantitative declines in recruitment globally; a synthesis of standardized data from 1974 until 2012 suggests that coral recruitment has been reduced by over 80% across the tropics (Price et al. 2019). The recent reduction of coral recruitment may be driven either by decline in settlement rate or reduction in survivorship of new settlers (*e.g.*, via declines of available adult brood stock) (Hughes et al. 2019). While estimates of settlement are available (Bak and Engel 1979; Price et al. 2019), we currently lack a quantitative description of the entire coral recruitment process, specifically the pattern and rate of demographic transition from juvenile to adult.

For reef-building corals, relatively little is known about the age at which the demographic rates of new arrivals become comparable to those of adults, thus defining the time scale of transition from settler to adult (i.e., the process of demographic recruitment). Studies of the earliest life stages of corals are particularly difficult in natural settings due to the challenge of detecting recently settled individuals and tracking them consistently through time (Vermeij and Sandin 2008). As a result, the demographics of early life stages have been limited largely to artificial substrata located in situ (Guerrini et al. 2020; Price et al. 2019) or in experimental settings (Harrington et al. 2004; Ritson-Williams et al. 2016), and usually over limited spatial and temporal scales. From such studies, it can be concluded that settling corals generally require 1 to 2 years to reach the juvenile stage, with the term ‘juvenile’ referring to young colonies with a maximum diameter greater than 1 cm, but less than 5 cm (Bak and Engel 1979; Doropoulos et al. 2015). Quantifying the density of juveniles is a common metric in coral population assessments, because such colonies are generally assumed to be indicators of near-future additions to the adult population (Miller et al. 2000; Trapon et al. 2013). However, a large body of work has demonstrated that coral survivorship is largely size-specific (Jackson and Hughes 1985; Kodera et al. 2020; Sebens 1987), thus we may expect survivorship of coral to change during and after this ‘juvenile’ phase (Babcock and Mundy 1996; Penin et al. 2010; Raymundo and Maypa 2004). With this background, it remains unclear how extensive the early life history bottleneck is and how long a coral remains as a juvenile before completing the process of demographic recruitment to the adult population.

The present study uses a large-area imaging approach to find and track juvenile corals annually at Palmyra Atoll from 2013 until 2017. The goal is to quantify survivorship patterns during this early life stage by estimating natural survival patterns over an extended spatial and temporal scale. We apply these time-series data to determine (1) the expected longevity, and rate of survivorship of juvenile corals through time, and (2) the functional form of coral survivorship (i.e., constant or logistic), considering potential influence of different sub-habitat types. The study spans multiple coral taxa, providing novel insights into the demographic distinctions among corals.

## Materials and methods

### Study site

**Fig. 1:**
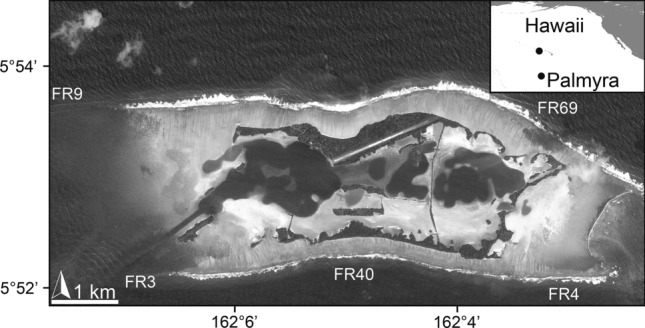
Location of Palmyra Atoll in the Central Pacific Ocean (inset), approximately 1600 km SW of Oahu, Hawaii. The plots (FR3, FR4, FR40, FR9, FR69) used in this study were initially distributed around the atoll on the 10-m depth, fore reef habitat

All fieldwork was conducted at Palmyra Atoll (5°52' N, 162°06' W) located in the Northern Line Islands in the Central Pacific Ocean (Fig. 1). Palmyra affords the opportunity to investigate demographics of a typical Pacific coral community in the relative absence of local anthropogenic stressors. Aside from the US military occupation during WWII, Palmyra has remained uninhabited or lightly inhabited throughout its history; in 2001 Palmyra was designated a US Fish and Wildlife Service National Wildlife Refuge with strict restrictions on resource use.

### Collection of large-area imagery

We used image-based mapping tools to identify juvenile corals and to track colony fates through time. In 2013, a series of 100 m^2^ plots were established around the atoll on the fore reef habitat at the 10-m depth contour, a habitat which is consistently available and often supports a high diversity and cover of corals (Russ 1984; Van Den Hoek et al. 1975), five of which were used for the present study (Fig. 1). The area within each plot was imaged annually in September–October, from 2013 to 2017, providing the source imagery needed to construct large-area imagery products (e.g., 3D models, orthophotomosaics). Notably, there were no widespread disturbance events affecting the coral community of Palmyra during this period (e.g., no anomalous wave activity, no predator outbreaks, and no widespread mortality linked to marine heat waves; Fox et al. 2019).

The details of the image collection process have been described previously (Kodera et al. 2020; Sandin et al. 2020a). Briefly, a diver-operated dual Nikon d7000 camera system was swum in a gridded pattern approximately 1–1.5 m from the bottom, collecting ~ 2500 images per camera in the course of a single SCUBA dive. One camera was set to a wide angle 18-mm focal length to ensure sufficient overlap for model construction, while the second camera was set to a 55-mm focal length to capture high resolution images of the benthos used for taxonomic identification and detection of small juvenile corals. All plots were established with georeferenced steel pins to facilitate repeated surveys.

### Large-area image creation

Large-area imagery refers to a suite of composite digital representations resulting from computer reconstructions of multiple images of natural scenes. Here, we generate three-dimensional (3D) reconstructions, estimated as point clouds, through Structure-from-Motion (SfM) algorithms (Westoby et al. 2012) using *Metashape* (Agisoft LLC., St. Petersburg, Russia). We then use the custom visualization and analytical platform *Viscore* (Fox et al. 2019; Sandin et al. 2020a) for referencing and data extraction. Scale was assigned by inputting the known distance between targets on 50-cm scale bars deployed in the field. To follow colonies through time without error introduced from shifting scale and orientation, all models from the time series are interactively coregistered in the *Viscore* program using fixed markers and invariant landscape features (Sandin et al. 2020a).

### Ecological post-processing

The current study builds off of a dataset used to describe spatial patterns of juvenile corals at Palmyra Atoll in 2013, following similar methods (Pedersen et al. 2019). Juvenile corals were defined as colonies with a maximum diameter greater than 1 cm and less than 5 cm in 2013. Based on previous evidence, it is likely that individuals in this size class represent a mixed-age cohort, including individuals having settled anywhere from a few months to, in the extreme, over 3 years prior to our first survey (Bak and Engel 1979; Doropoulos et al. 2015; Kayal et al. 2018). Taxonomic identifications were made to the genus level and included only those individuals which could be identified reliably as sexual recruits, as opposed to fragments or remnant tissue from existing adult colonies (Online Resource, Table S1). All taxa from Pedersen et al. (2019) were included in this study with the exception of corals from the family *Fungiidae*, given that adult colonies of this taxon are not physically attached to the substrate, thus preventing reliable relocation and tracking of colony fate.

**Fig. 2:**
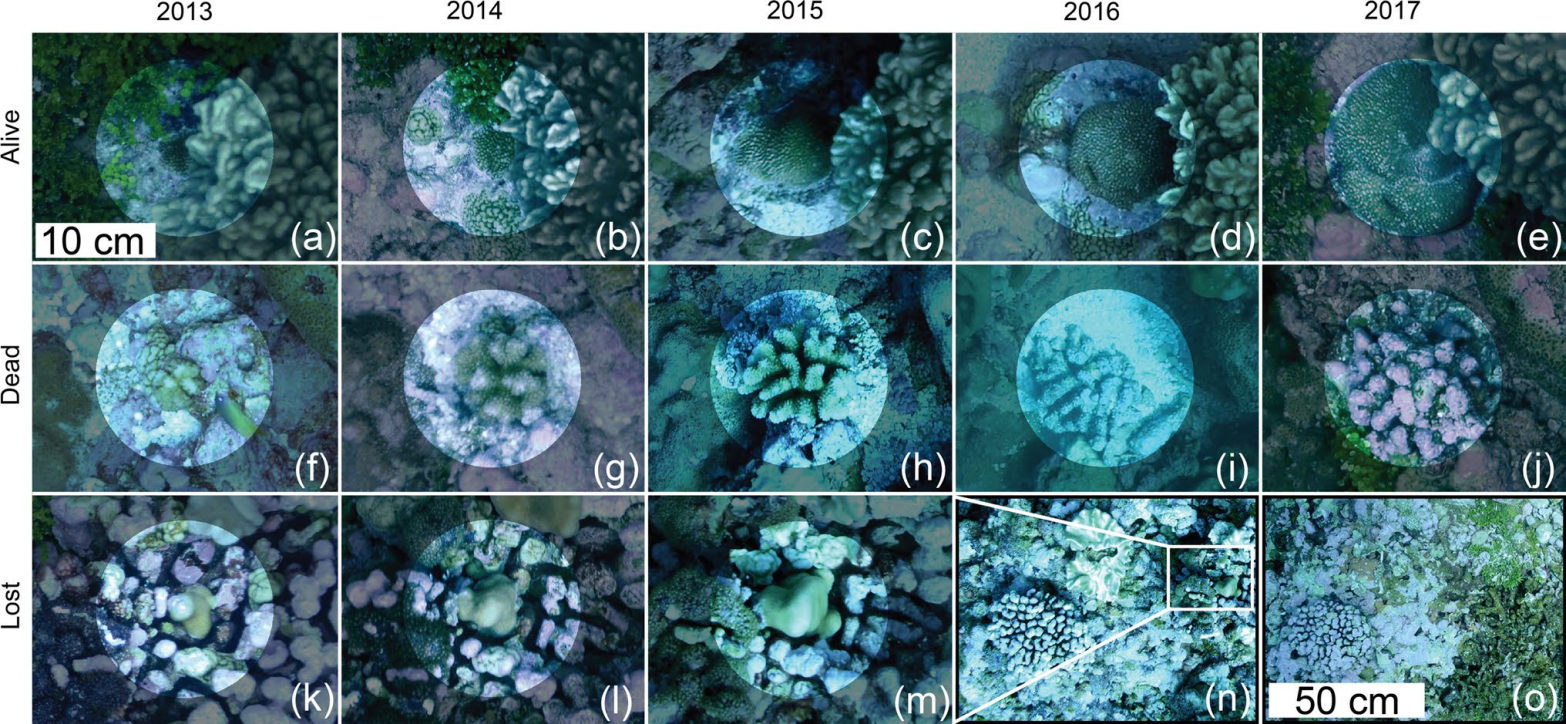
Life series representing examples of each possible juveniles’ fate through time, being “Alive” (i.e., staying alive until the end of the survey), “Dead” (i.e., dying during the survey) or “Lost” (i.e., having died as a result of being dislodged at some point during the survey). Scale for insets **a**–**m** is provided in inset (**a**), scale for insert (**n**–**o**) is provided in inset (**o**). The white circled boxes (**a**–**m**) show photos of juvenile colonies, located at the circles’ center, while the black circled boxes (**n**–**o**) show locations of the reefs. **a**–**e** A *Hydnophora* juvenile colony is shown growing through time, from 2013 to 2017, and is still alive at the end of the survey. **f**–**j** A *Pocillopora* juvenile colony is shown growing from 2013 to 2015, then it shrank and lost live tissue in 2016, to be found dead (covered in crustose coralline algae) in 2017. **k**–**m** A *Pavona* juvenile colony is shown growing from 2013 to 2015. **n** The photo shows the location of the *Pavona* colony in 2015, with a *Pocillopora* colony easily recognizable and used as landmark. **o** The same location in the reef is shown at the subsequent year, 2016, where the *Pocillopora* colony is still present but the surroundings have undergone disturbances and all signs of the *Pavona* juvenile coral are gone

All juvenile corals identified in the 2013 orthophotomosaics from five sites around Palmyra (Fig. 1) were located and mapped onto 3D point clouds in *Viscore*. With data in virtual 3D maps, fate-tracking of individual colonies through the layers of coregistered, annually collected point clouds was completed. To improve visualizations of individual colonies represented in the point cloud, the source imagery containing the focal colony could be referred to using the image overlay feature in *Viscore* (for more information please see online supplemental materials in Fox et al. 2019). Colonies alive in 2013 were tracked in each subsequent annual survey and assigned to one of three categories: (1) alive (living tissue present, including bleached colonies and those that had lost tissue from the previous time point but were still alive), (2) dead (the skeleton was found at the exact same location but without any visible remaining living tissue) and (3) lost (no trace of the colony could be found) (Fig. 2). For coral colonies in the size class of interest, the ‘lost’ category equates to mortality, albeit through a distinct means of death (physical dislodgement resulting in death of ‘lost’ individuals *vs.* the myriad factors causing death through loss of live tissue among ‘dead’ individuals). Although natural recruitment occurred throughout the study, we focused only on the cohort of colonies initially located in 2013. The substrate underneath each colony was identified as unconsolidated (any areas of loose reef, often dead branches or colonies that had broken off from the substrate and had no evidence of encrusting and calcifying organisms that had begun to cement loose pieces down, including rare sand areas) or consolidated (all other areas), based on degree of small-scale structural changes through time observed and mapped onto each model (Pedersen et al. 2019).

Patterns of longevity were computed over a 4-year sampling window (2013–2017) as the proportion of juvenile colonies identified from 2013 that died within each annual time window, defined as ‘0’, ‘1’, ‘2’, ‘3’ and ‘ ≥ 4’, reflecting the (truncated) integer number of years colonies survived. For instance, the longevity value in ‘0’ is the proportion of juveniles identified in 2013 that survived less than 1 year and the value in ‘2’ is the proportion that survived at least 2 years and did not survive until year 3. The final category of ‘ ≥ 4’ represents all individuals that were identified from 2013 that survived through the duration of the 4-year study window. As a result, in those cases where early mortality rates are low, the ‘ ≥ 4’ category will include a large proportion of the originally identified individuals.

### Statistical analysis

Analyses were conducted to estimate patterns of survivorship for juvenile corals through time. Given that surveys were conducted once annually, we define survivorship as a function of time, *s*_*t*_, describing the annual probability of survivorship from year *t*-1 until *t* for *t* > 0 (*s*_*0*_ is defined with a value of 1, given that the colony had to have survived to have been observed at initial survey, time point 0). We contrasted the performance of two nested models describing survivorship with time: (i) constant survivorship, defined with the single constant through time (${s}_{t}={s}_{c}$), or (ii) logistic survivorship (${s}_{t}=s/\left(1+{e}^{-bt}\right)$, where *t* is a positive integer, and *s* and *b* are estimated constants. We assumed that the fate of each individual was independent of all others, consistent with a binomial error structure.

Best-fit parameters of the survivorship models were estimated through maximum likelihood estimation (MLE). Given that the survivorship models describe the annual probability of surviving from 1 year to the next, the probability of surviving multiple years will be product of multiple annual survivorship probabilities. For example, the probability of a colony being found dead in the third year will be the product of surviving from year 0 to 1, from year 1 to 2, and then the probability of dying from year 2 to 3 (calculated as 1 minus the probability of surviving from year 2 to 3). The probability of surviving until time *t* and dying before time *t* + 1 (defined as a longevity of *t*) is expressed generally as $\left(1-{s}_{t+1}\right){\prod }_{i=0}^{t}{s}_{i}$, where the values of *s*_*i*_ are defined by the survivorship models. The calculation of likelihood based upon one particular survivorship model (with candidate parameters) for one set of survivorship data (i.e.*,* a vector of colony-specific longevity values for all colonies within the data grouping) will thus be the estimated probability (log-transformed) of surviving to the specified age realized by a colony, summed across all colonies within the grouping. MLE (using the Nelder–Mead method) was used to identify the best-fit parameters for each survivorship model for each taxonomic grouping.

The best-fit models were selected by comparing survivorship models across data groupings by habitat. First, we contrasted the relative fit of the constant and logistic survivorship models across all benthic surfaces (Models 1 and 2, respectively). Second, to evaluate the potential importance of habitat type (i.e., consolidated, unconsolidated) on survivorship patterns, independent models were fit for juveniles living on each habitat type. The four combinations of models (constant vs logistic for each unconsolidated and consolidated habitats) were evaluated as Models 3–6 (Table 1). To ensure significant statistical power in the investigations of single taxa, a minimum sample size of ten colonies total was required for Models 1 and 2. For the two habitat models (Models 3–6), a minimum of five colonies per habitat was required.

**Table 1:** Comparison among models using Akaike’s Information Criteria (AIC) based on maximum likelihood results for coral juveniles by taxonomic grouping

Models	*n*	Taxa									
		All corals	*Pocillopora*	*Stylophora*	*Pavona*	*Goniastrea*	*Hydnophora*	*Astrea*	*Porites*	*Acropora*	*Favites*
One habitat											
Constant (1)	1	1507.8	487.2	337.8	165.0	121.4	105.6	**98.4**	43.0	43.8	33.4
Logistic (2)	2	1428.2***	478.6**	324.0***	147.8***	113.8**	94.0***	99.8	43.2	**42.0** *	**29.8** *
Two habitats (unconsolidated + consolidated)											
Constant + constant (3)	2	1509.4	489.2	339.6	167.0	123.4	–	100.2	**42.2**	–	–
Constant + logistic (4)	3	1438.0	481.6	331.6	149.0	118.8	–	99.4	42.8	–	–
Logistic + constant (5)	3	1503.4	490.2	335.4	167.8	122.2	–	102.2	44.0	–	–
Logistic + logistic (6)	4	1432.0	482.6	327.4	149.8	117.8	–	101.4	44.8	–	–

Models are numbered and organized by habitat type and survival assumptions (*i.e.*, constant *vs* logistic, one *vs* two habitat types). *n* is the number of parameters per model. The best-supported model, with the lowest AIC value, is indicated in bold; alternative models that were indistinguishable from the best-supported model (ΔAIC < 2) are underlined. The AIC analysis for the two-habitat models (3–6) was not run for *Hydnophora*, *Acropora* and *Favites* (grey parts) as the taxa failed to reach the threshold of at least five individuals by habitat type

**Fig. 3:**
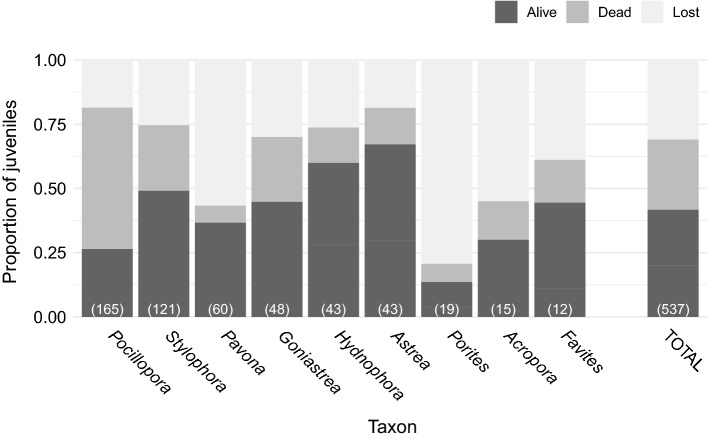
Final summary of fates of coral juveniles tracked over the 4-year window (2013–2017). Demographic fate is separated into three categories – ‘Alive’ (colony surviving), ‘Dead’ (live tissue gone with skeleton remaining), and ‘Lost’ (colony dislodged and gone from original location and also considered dead). Data are organized by taxon (ordered by decreasing initial abundance), with initial abundance in 2013 reported in the bottom of each bar. The ‘TOTAL’ bar indicates the proportions for colonies of all taxa pooled together

Results of model comparison described in Fig. 3, testing relative fit of logistic model (2) relative to simpler constant model (1) assuming homogeneous survival patterns across habitats (‘one habitat’), are reported with asterisks (*p < 0.05; **p < 0.01; ***p < 0.001; NS denotes lack of significance)

Likelihood ratio tests (LRT) were used to compare models of constant and logistic annual survival across habitats (Models 1 and 2, respectively). Because Models 3–6 are not each nested among one another and with Models 1–2, Akaike’s Information Criterion (AIC) was used instead of LRT to evaluate relative model fit. The AIC for a model $i$ was computed as [$-2 \mathrm{ln}\left({L}_{i}\right)+2{n}_{i}]$, where ${L}_{i}$ is the likelihood value for the best-fit parameters of Model $i$ and ${n}_{i}$ is the number of parameters in the model. The model with the lowest AIC values was taken as the best supported model, and models with an AIC difference of less than two relative to the best supported model were considered to have comparable statistical support.

All statistical analyses were performed using R (version 3.5.2) statistical software (R Development Core Team 2018).

## Results

In 2013, a total of 537 juveniles from 12 taxa were identified and mapped across the 5 100-m^2^ plots (Fig. 3; Online Resource, Tables S1 and S2). The abundance of juveniles varied by taxon, ranging from 165 colonies of *Pocillopora*, to only 3 *Leptastrea* colonies. After 4 years, 40.8% of all juveniles were still alive, 30.3% had died due to complete tissue loss, and the remaining 28.9% died after being lost to dislodgement (Fig. 3; Online Resource, Table S1). Among taxa, *Astrea* had the highest proportion of survivors (65%), and *Porites* had the lowest (11%). There were differences among taxa in the proportion of mortality attributed to the loss of colonies. In *Pocillopora*, most mortality was due to colonies that experienced death (54%), with far fewer colonies (19%) dying as a result of becoming dislodged and lost over the course of the study. In contrast, several taxa experienced greater mortality due to loss than to death, including *Pavona*, *Porites*, *Acropora*, and *Favites* (Fig. 3; Online Resource, Table S1). Across sites, consolidated habitat was predominant, comprising 82.8% (± 2.1 SE) of the planar area, and a similar proportion of juveniles were found in this habitat type (81%).

**Fig. 4:**
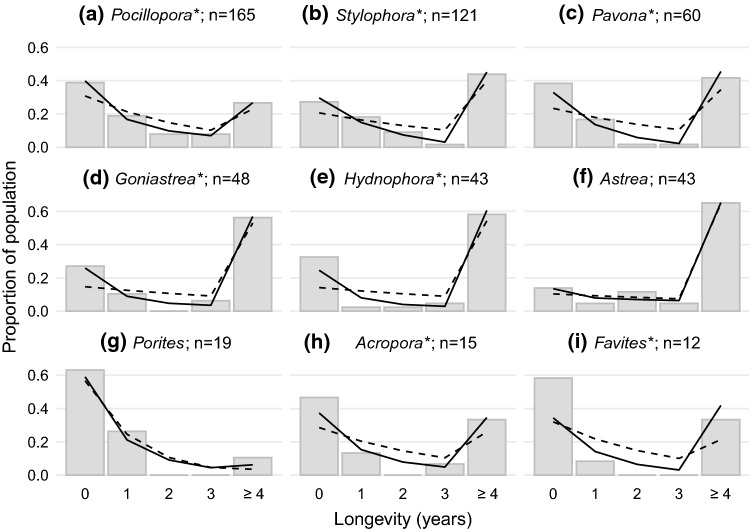
Patterns of longevity for coral juveniles of nine taxa over a 4-year sampling window (2013–2017). The distribution of realized longevity across the population is reported with bars, each representing the proportion of starting individuals (identified in 2013) that died within the time step window (*e.g.*, value in ‘0’ is the proportion of colonies that survived less than one year after the initial survey, value in ‘2’ is the proportion that survived until the year 2 sampling period but did not survive until year 3). All colonies that survived through the last sampling interval are binned in ‘ ≥ 4’, reflecting that these colonies had a longevity of 4 or more years. Consequently, survivorship in this final interval will often appear large relative to the others as it represents the proportion of colonies first surveyed in 2013 that were still alive in 2017, and thus represents all colonies with longevities of 4 or greater. Lines present the statistical expectation for the proportion of the colonies expected to fall into each longevity class based upon the two survivorship models. Lines are estimated based upon best-fit parameters for models of constant annual survival (dashed lines) and logistic annual survival (solid lines). The proportions of colonies estimated to survive until time *t* are calculated as $\left(1-{s}_{t+1}\right){\prod }_{i=0}^{t}{s}_{i}$, where *s*_*t*_ is defined by the survivorship models. As such, the estimated longevity at each year is the cumulative probability of surviving each time point before and not surviving until the next year. The estimated longevity in the final time point of ‘ ≥ 4’ reflects the cumulative probability of surviving to at least 4 years (including all subsequent longevities). Models reported are for a single habitat type, and asterisks following the genus name indicate statistical support (*p* < 0.05) of the logistic model relative to the constant model using a likelihood ratio test

Most taxa showed increases in annual survivorship through time. Across habitats, and considering all corals combined, model comparison revealed that the data were better fit by a logistic, relative to constant, model of survivorship (Model 2 preferred to Model 1; *χ*^2^ value = 81.7, *P* < 0.01) (Online Resource, Figure S5a), with a positive estimate of *b* indicating that the probability of survivorship increases through time (Online Resource, Table S3). In general, adding consideration of habitat type did not improve estimates of survival patterns for juvenile corals (Table 1). For all corals pooled together, the one-habitat logistic model (2) was selected as the best-fit among our set of six candidate models (Models 1–2 without habitat effect; Models 3–6 with habitat effects; Table 1). When taxa were investigated individually, we found consistent support for Model 2 for all taxa except *Astrea* and *Porites*, which were best supported by Model 1 (equivalent constant survival across habitats) and Model 3 (constant survival with rate specific to each habitat), respectively (Fig. 4; Online Resource, Table S3).

The estimated survival through time over the 4-year interval (based upon best model fit), with extrapolation out to 10 years, is shown for all corals (Online Resource, Figure S5b) and by taxon (Online Resource, Figure S6). Among those taxa demonstrating logistic survivorship, *Pocillopora*, *Goniastrea* and *Hydnophora* are predicted to reach within 99% of maximum annual survival within the 4-year survey, with the four remaining taxa also achieving high survivorship (> 80% per annum) by the end of the survey. Of the taxa supported by models of constant survivorship, *Astrea* revealed a consistently high value (90%), while *Porites* had a notably low value (43%).

## Discussion

This study offers insights into the survival patterns of juvenile corals, providing estimates of the duration of pre-adult life stages for coral taxa common in the tropical Pacific. Over the course of the study, 40.8% of the initial cohort of juveniles survived for the full 4 years; if one were to assume a constant annual rate of survival, the approximate mean annual survivorship would be 80%. While comparisons with other studies of juvenile coral survivorship should be made with caution due to notable differences in methodology and taxonomic breadth, our results are consistent with other published survival estimates which range from 27 to 88% (data from studies converted to annual survival assuming constant rates; Bak and Engel 1979; Bongiorni et al. 2011; Connell 1973; Doropoulos et al. 2015; Trapon et al. 2013; Villanueva et al. 2012). Notably, these studies tracked survival for a shorter period (ranging from 6 to 24 months), hence describing survivorship most comparable to the first time window of this study. The data presented in this study offer an additional form of information on juvenile survival, notably tracking colonies individually across multiple years. Through such longitudinal sampling, it becomes possible to understand more about age-dependence of early survivorship patterns of coral, contributing insights to define quantitatively the demographic process of recruitment to the adult population.

Across ecosystems and groups of organisms, the process of recruitment for a cohort is not considered complete until individuals have passed through important developmental transitions and the survival rate of early-life stage individuals approaches that observed among adults. While definitions vary, most authors consider corals with maximum diameter of 1–5 cm as belonging to the juvenile life stage (Edmunds 2007; Miller et al. 2000; Trapon et al. 2013). Importantly, the term “juvenile coral” has been applied most commonly to the stock of individuals thought to have completed the recruitment process but not yet reached sexual maturity (Bak and Engel 1979; Miller et al. 2000). Such an operational definition suggests that juveniles have passed through the survivorship bottleneck of the early life history phase to reach adult-like demographic rates. Given that coral demography is notably size-dependent (Doropoulos et al. 2012), however, it is likely that the juvenile phase may not fully share demographic expectations of the adult stock (Hughes 1984; Kodera et al. 2020). By tracking a cohort of individuals through time, we were able to explore quantitative evidence of demographic shifts with age for juvenile corals, contrasting survivorship models without and with age-dependence (constant vs logistic survivorship with time, respectively). We found overwhelming support for models of logistic survivorship among juvenile corals, supporting the hypothesis that reaching the juvenile class (1–5 cm maximum diameter) is not synonymous with escaping the early life stage survivorship bottleneck.

For the study system described here, we find that the transitional juvenile stage for most taxa ended 3–4 years after reaching the 1–5 cm diameter size class (the size at which they were identified in the first time point). Notably, the age of colonies within the 1–5 cm diameter size class is not clear; better estimated are rates of colony growth which we can use to provide some bounds on likely age. Estimations from forereef habitats on Moorea, for example, suggest that the majority of colonies in this size range (> 90%) will grow out of the size range within 1 year (Kayal et al. 2018), while comparable assessment of colony-specific growth in reef flat and forereef habitats of the Great Barrier Reef suggest that colonies in this approximate size range may take upwards of 3 years to grow into the next bigger size class (Doropoulos et al. 2015). As such, we may assume that colonies within this size class range in age from < 1 year to over 3 years post settlement (Bak and Engel 1979; Doropoulos et al. 2015; Kayal et al. 2018). When combined with the survivorship data here, we can estimate that a recently settled larva will complete the demographic process of recruitment into the adult population after somewhere in the range of 3–7 years. While the timeline to complete recruitment was similar for most taxa, there were large differences in the maximum annual survivorship that was achieved by the end of the study (< 50% to > 95%). Extrapolation of these survival rates to other systems, or beyond 4 years, should be done with caution as they do not take into account episodic or severe disturbances (*e.g.,* diseases, competition, heat waves, cyclones).

The survival patterns of early life history corals and preference for settlement substrate for newly arriving larvae to a reef is driven by a number of physical and biotic factors, including exposure (e.g., crevices; Brandl et al. 2014; Roth and Knowlton 2009), biological coverage (e.g., taxa of crustose coralline algae; Harrington et al. 2004), and specifics of biofilm composition (Webster et al. 2004). Evidence suggests that early life stage survivorship patterns result in spatial distributional patterns at later stages of development. On Palmyra, Pedersen et al. (2019) noted a non-random association of juveniles from a number of taxa, showing overrepresentation on unconsolidated substrates (with only *Pocillopora* showing overrepresentation on consolidated substrates). Importantly, in our plots, unconsolidated substrata are largely composed of semi-consolidated chunks of coral skeletons (largely from branching coral taxa), typically > 20 cm in maximum diameter, and vulnerable to physical tumbling only during the larger swell events (e.g., annual winter swell events typical to the central Pacific). This definition of unconsolidated substrate is distinct from the smaller ‘rubble’ common to many reefs, which is composed of < 10 cm fragments of skeletons and very unstable under even modest wave energy. Using this definition of unconsolidated substrate, we found here that the patterns of survival over 4 years for individual juveniles were largely unaffected by the colony’s substrate (Table 1). These data suggest that the episodic rearrangement of substrate has limited impact on the realized demographic rates of juvenile corals, supporting the hypothesis that unconsolidated substrate may be a transitional habitat that is in the process of being re-consolidated through the growth and calcification of corals and other calcifying organisms (Pedersen et al. 2019).

Compositional change in coral communities as a result of dislodgment and loss of colonies from storm induced damage (i.e., waves) is well known and has been widely studied (Madin and Connolly 2006; Massel and Done 1993; Sandin et al. 2020b). *Pocillopora* in particular experienced mortality almost exclusively through loss of live tissue from an intact skeleton which is in contrast to most other taxa, especially *Porites,* which suffered substantial mortality due to dislodgement (Fig. 3). It is notable that *Pocillopora* is the only taxon shown to be overrepresented on consolidated substrates as juveniles, with other studied taxa showing non-random preference for unconsolidated substrate (Pedersen et al. 2019). While the rate of mortality does not show strong association with substrate type, these results suggest the cause of mortality may be linked to substrate, leading to intriguing hypotheses regarding development strategies and skeletal investments based upon settlement preference across coral taxa.

## Conclusions

The present study provides key insights into natural processes and dynamics that take place in an early life stage of reef-building corals. Using a 4-year time series of annual sampling, we demonstrate that juvenile corals generally show an increasing and saturating rate of annual survival with time. Most taxa reach an estimated maximum annual survival after 3 or more years, suggesting that the process of demographic recruitment in corals can be estimated to occur 4–6 years after settlement for many Pacific taxa. Efforts of coral management, including monitoring and restoration, are emphasizing the growing importance of maximizing recruitment of sexually produced colonies to the adult population. This study provides needed information for the design of monitoring efforts and the interpretation of results, helping to contextualize the time scale associated with adult replenishment among coral communities.

## Supplementary Information

Below is the link to the electronic supplementary material.

Supplementary file1 (PDF 937 kb)

## Data Availability

Data and relevant code for this research work are stored in GitHub: https://github.com/LSarribouette/Juvenile-paper-2020.
