# Supplementary material for: Post-settlement demographics of reef building corals suggest prolonged recruitment bottlenecks
Source: Oecologia. 2022 Jun 4;199(2):387–96. doi: 10.1007/s00442-022-05196-7 (PMC9226083; doi:10.1007/s00442-022-05196-7)
Supplement: Supplementary file 1 — Supplementary file1 (PDF 937 kb) [file 442_2022_5196_MOESM1_ESM.pdf]

**Post-settlement demographics of reef building corals suggest prolonged recruitment bottlenecks**

Lauranne Sarribouette[1], Nicole E. Pedersen[1], Clinton B. Edwards[1], Stuart A. Sandin[1,*]

[1] Scripps Institution of Oceanography, UC San Diego, La Jolla, CA, USA

[*]corresponding author, email: ssandin@ucsd.edu; Phone: +1 (858) 534-4150, Fax: +1 (858) 822-1267

Table S1: Abundance and survival of juveniles per taxon, in each time window.

| Taxa | Initial abundance | Transition | | | | | | | | | | | | Survival (%) |
|---|---|---|---|---|---|---|---|---|---|---|---|---|---|---|
| | | *1* | | | *2* | | | *3* | | | *4* | | | |
| | | Alive | Dead | Lost | Alive | Dead | Lost | Alive | Dead | Lost | Alive | Dead | Lost | |
| *Pocillopora* | 165 | 101 | 49 | 15 | 70 | 67 | 28 | 57 | 79 | 29 | 44 | 89 | 32 | 27 |
| *Stylophora* | 121 | 88 | 12 | 21 | 66 | 24 | 31 | 55 | 35 | 31 | 53 | 37 | 31 | 44 |
| *Pavona* | 60 | 37 | 2 | 21 | 27 | 5 | 28 | 26 | 5 | 29 | 25 | 6 | 29 | 42 |
| *Goniastrea* | 48 | 35 | 5 | 8 | 30 | 8 | 10 | 30 | 8 | 10 | 27 | 10 | 10 | 56 |
| *Hydnophora* | 43 | 29 | 3 | 11 | 28 | 3 | 12 | 27 | 4 | 12 | 25 | 5 | 13 | 58 |
| *Astrea* | 43 | 37 | 0 | 6 | 35 | 2 | 6 | 30 | 5 | 8 | 28 | 7 | 8 | 65 |
| *Porites* | 19 | 7 | 1 | 11 | 2 | 2 | 15 | 2 | 2 | 15 | 2 | 2 | 15 | 11 |
| *Acropora* | 15 | 8 | 0 | 7 | 6 | 1 | 8 | 6 | 1 | 8 | 5 | 2 | 8 | 33 |
| *Favites* | 12 | 5 | 2 | 5 | 4 | 3 | 5 | 4 | 3 | 5 | 4 | 3 | 5 | 33 |
| *Turbinaria* | 5 | 4 | 0 | 1 | 3 | 1 | 1 | 3 | 1 | 1 | 3 | 1 | 1 | 60 |
| *Platygyra* | 3 | 3 | 0 | 0 | 3 | 0 | 0 | 3 | 0 | 0 | 3 | 0 | 0 | 100 |
| *Leptastrea* | 3 | 2 | 0 | 1 | 1 | 0 | 2 | 0 | 0 | 3 | 0 | 0 | 3 | 0 |
| Total | 537 | 356 | 74 | 107 | 275 | 116 | 146 | 243 | 143 | 151 | 219 | 163 | 155 | 41 |

*Notes:* Taxa are ordered by decreasing initial abundance.

Table S2: Abundance of juveniles per taxon separated by habitat types, in each time window.

| Taxa | Habitat | Initial abundance | Transition | | | | | | | | | | | |
|---|---|---|---|---|---|---|---|---|---|---|---|---|---|---|
| | | | *1* | | | *2* | | | *3* | | | *4* | | |
| | | | Alive | Dead | Lost | Alive | Dead | Lost | Alive | Dead | Lost | Alive | Dead | Lost |
| *Pocillopora* | Cons | 128 | 77 | 39 | 12 | 54 | 53 | 21 | 45 | 62 | 21 | 34 | 71 | 23 |
| | Uncons | 37 | 24 | 10 | 3 | 16 | 14 | 7 | 12 | 17 | 8 | 10 | 18 | 9 |
| *Stylophora* | Cons | 92 | 67 | 11 | 14 | 50 | 19 | 23 | 41 | 28 | 23 | 39 | 30 | 23 |
| | Uncons | 29 | 21 | 1 | 7 | 16 | 5 | 8 | 14 | 7 | 8 | 14 | 7 | 8 |
| *Pavona* | Cons | 49 | 30 | 2 | 17 | 21 | 4 | 24 | 21 | 4 | 24 | 21 | 4 | 24 |
| | Uncons | 11 | 7 | 4 | 1 | 6 | 1 | 4 | 5 | 1 | 5 | 4 | 2 | 5 |
| *Goniastrea* | Cons | 34 | 25 | 3 | 6 | 21 | 5 | 8 | 21 | 5 | 8 | 19 | 7 | 8 |
| | Uncons | 14 | 10 | 2 | 2 | 9 | 3 | 2 | 9 | 3 | 2 | 8 | 4 | 2 |
| *Hydnophora* | Cons | 40 | 26 | 3 | 11 | 25 | 3 | 12 | 24 | 4 | 12 | 22 | 5 | 13 |
| | Uncons | 3 | 3 | 0 | 0 | 3 | 0 | 0 | 3 | 0 | 0 | 3 | 0 | 0 |
| *Astrea* | Cons | 34 | 28 | 0 | 6 | 27 | 1 | 6 | 24 | 2 | 8 | 23 | 3 | 8 |
| | Uncons | 9 | 9 | 0 | 0 | 8 | 1 | 0 | 6 | 3 | 0 | 5 | 4 | 0 |
| *Porites* | Cons | 14 | 3 | 1 | 10 | 1 | 2 | 11 | 1 | 2 | 11 | 1 | 2 | 11 |
| | Uncons | 5 | 4 | 0 | 1 | 1 | 0 | 4 | 1 | 0 | 4 | 1 | 0 | 4 |
| *Acropora* | Cons | 14 | 8 | 0 | 6 | 6 | 1 | 7 | 6 | 1 | 7 | 5 | 2 | 7 |
| | Uncons | 1 | 0 | 0 | 1 | 0 | 0 | 1 | 0 | 0 | 1 | 0 | 0 | 1 |
| *Favites* | Cons | 9 | 3 | 1 | 5 | 3 | 1 | 5 | 3 | 1 | 5 | 3 | 1 | 5 |
| | Uncons | 3 | 2 | 1 | 0 | 1 | 2 | 0 | 1 | 2 | 0 | 1 | 2 | 0 |
| *Turbinaria* | Cons | 4 | 3 | 0 | 1 | 2 | 1 | 1 | 2 | 1 | 1 | 2 | 1 | 1 |
| | Uncons | 1 | 1 | 0 | 0 | 1 | 0 | 0 | 1 | 0 | 0 | 1 | 0 | 0 |
| *Platygyra* | Cons | 1 | 1 | 0 | 0 | 1 | 0 | 0 | 1 | 0 | 0 | 1 | 0 | 0 |
| | Uncons | 2 | 2 | 0 | 0 | 2 | 0 | 0 | 2 | 0 | 0 | 2 | 0 | 0 |
| *Leptastrea* | Cons | 3 | 2 | 0 | 1 | 1 | 0 | 2 | 0 | 0 | 3 | 0 | 0 | 3 |
| | Uncons | 0 | 0 | 0 | 0 | 0 | 0 | 0 | 0 | 0 | 0 | 0 | 0 | 0 |
| Total | Cons | 422 | 273 | 60 | 89 | 212 | 90 | 120 | 189 | 110 | 123 | 170 | 126 | 126 |
| | Uncons | 115 | 83 | 18 | 15 | 63 | 26 | 26 | 54 | 33 | 28 | 49 | 37 | 29 |

*Notes:* Taxa are ordered by decreasing initial abundance. Habitat types are described as 'Cons' for consolidated areas and 'Uncons' for unconsolidated areas.

Table S3: Likelihood ratio test results of the comparison of one-habitat constant (1) and logistic (2) model, for all taxa pooled together, and by taxon.

| Group | $\chi^2$ value | | Best-fit model | Best-fit model parameters |
|---|---|---|---|---|
| All coral (Alive, Dead, Lost) | 81.7 | *** | Logistic | s=0.94 b=0.82 |
| All coral (Alive, Dead) | 16.7 | *** | Logistic | s=0.90 b=2.06 |
| *Pocillopora* | 10.6 | ** | Logistic | s=0.80 b=1.09 |
| *Stylophora* | 15.8 | *** | Logistic | s=1.23 b=0.29 |
| *Pavona* | 19.2 | *** | Logistic | s=1.04 b=0.59 |
| *Goniastrea* | 9.5 | ** | Logistic | s=0.95 b=1.28 |
| *Hydnophora* | 13.6 | *** | Logistic | s=0.96 b=1.30 |
| *Astrea* | 0.7 | NS | Constant | $s_c$=0.90 |
| *Porites* | 1.8 | NS | Constant | $s_c$=0.43 |
| *Acropora* | 3.9 | * | Logistic | s=0.92 b=0.78 |
| *Favites* | 5.5 | * | Logistic | s=1.01 b=0.63 |

*Notes:* Results correspond to statistical summaries in table 1 and figure 3. Taxa are ordered by decreasing initial abundance. The number of degrees of freedom is one for all comparisons. Asterisks indicate significance: * p<0.05; ** p<0.01; *** p<0.001; NS denotes lack of significance.

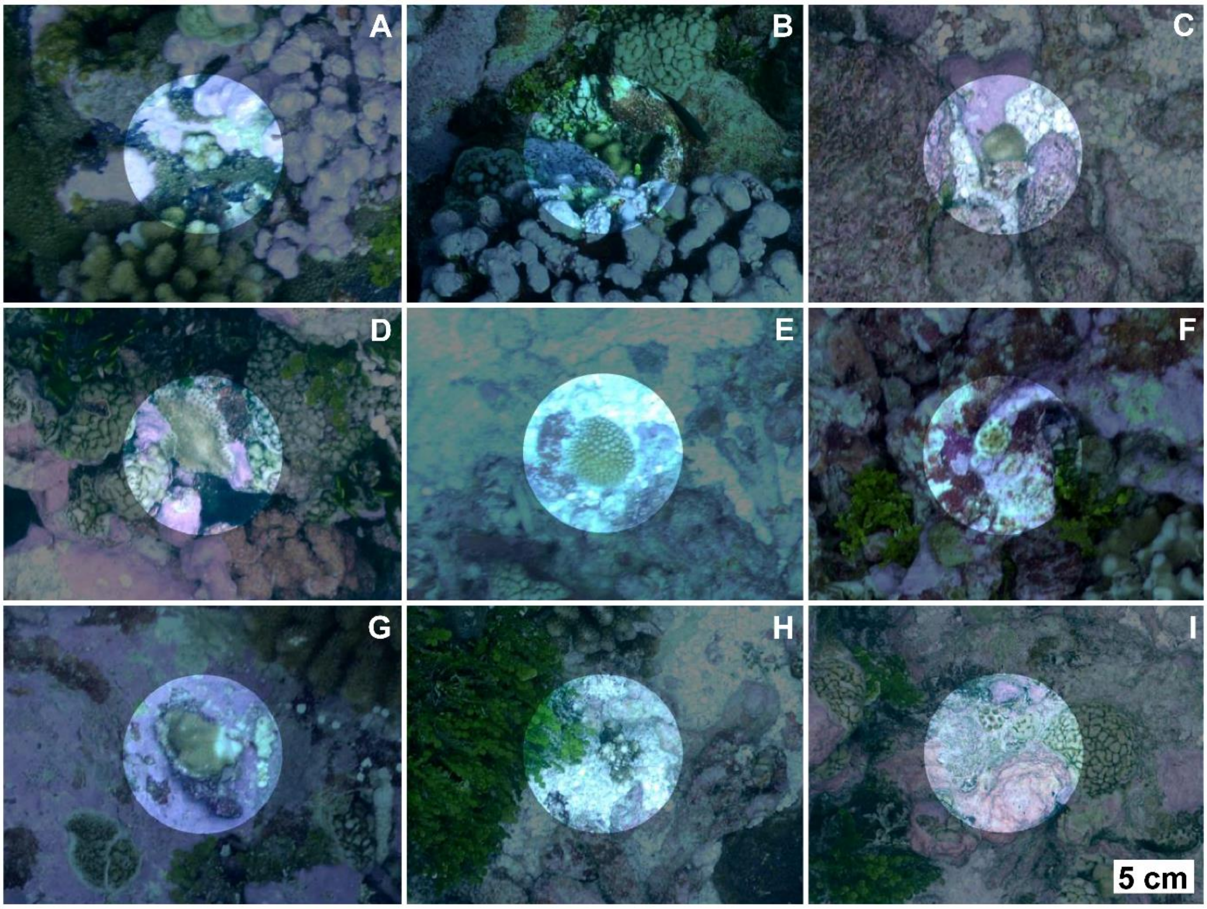


Figure S4: Photos of the nine taxa individually studied, taken on initial year (2013) and located at the center of each circle. Taxa are ordered by decreasing initial abundance, namely (A) *Pocillopora*, (B) *Stylophora*, (C) *Pavona*, (D) *Goniastrea*, (E) *Hydnophora*, (F) *Astrea*, (G) *Porites*, (H) *Acropora*, (I) *Favites*. The juvenile colonies' size range from 1-5cm.

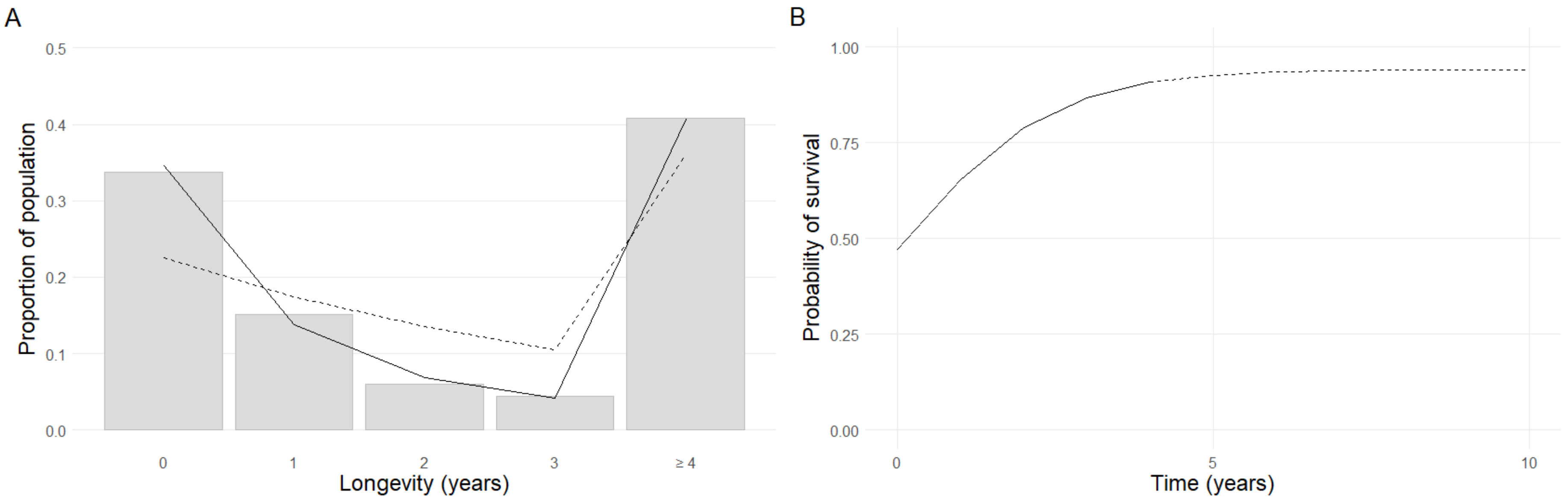


Figure S5: (a) Patterns of longevity for coral juveniles of all taxa over a 4-year sampling window (2013-2017). The distribution of realized longevity across the population is reported with bars, each representing the proportion of starting individuals (identified in 2013) that died or was lost within the time step window (*e.g.*, value in '0' is the proportion of colonies that survived less than one year, value in '2' is the proportion that survived at least 2 years and did not survive until year 3). All colonies that survived through the last sampling interval are binned in '≥4', reflecting that these colonies had a longevity of 4 or more years. Idealized longevity ($\hat{\boldsymbol{L}}_{\boldsymbol{t}}$, defined in text) is the statistical expectation of the proportion of the starting population predicted to be removed from the surviving population between time *t* and *t*+1, and is estimated based upon best-fit parameters for models of constant annual survival (dashed lines) and logistic annual survival (solid lines). Models reported are for a single habitat type, and asterisks following the genus name indicate statistical support ($p<0.05$) of the logistic model relative to the constant model using a Likelihood Ratio Test. (b) Estimated survival through time for colonies of all taxa pooled together. It was computed from statistical expectations based upon best-fit parameters (*i.e.*, s = 0.94 and b = 0.82) for a logistic model (2), assuming homogeneous survival patterns across habitats ('one habitat'). Solid lines represent the signal fitting the data, dashed lines represent further extrapolations.

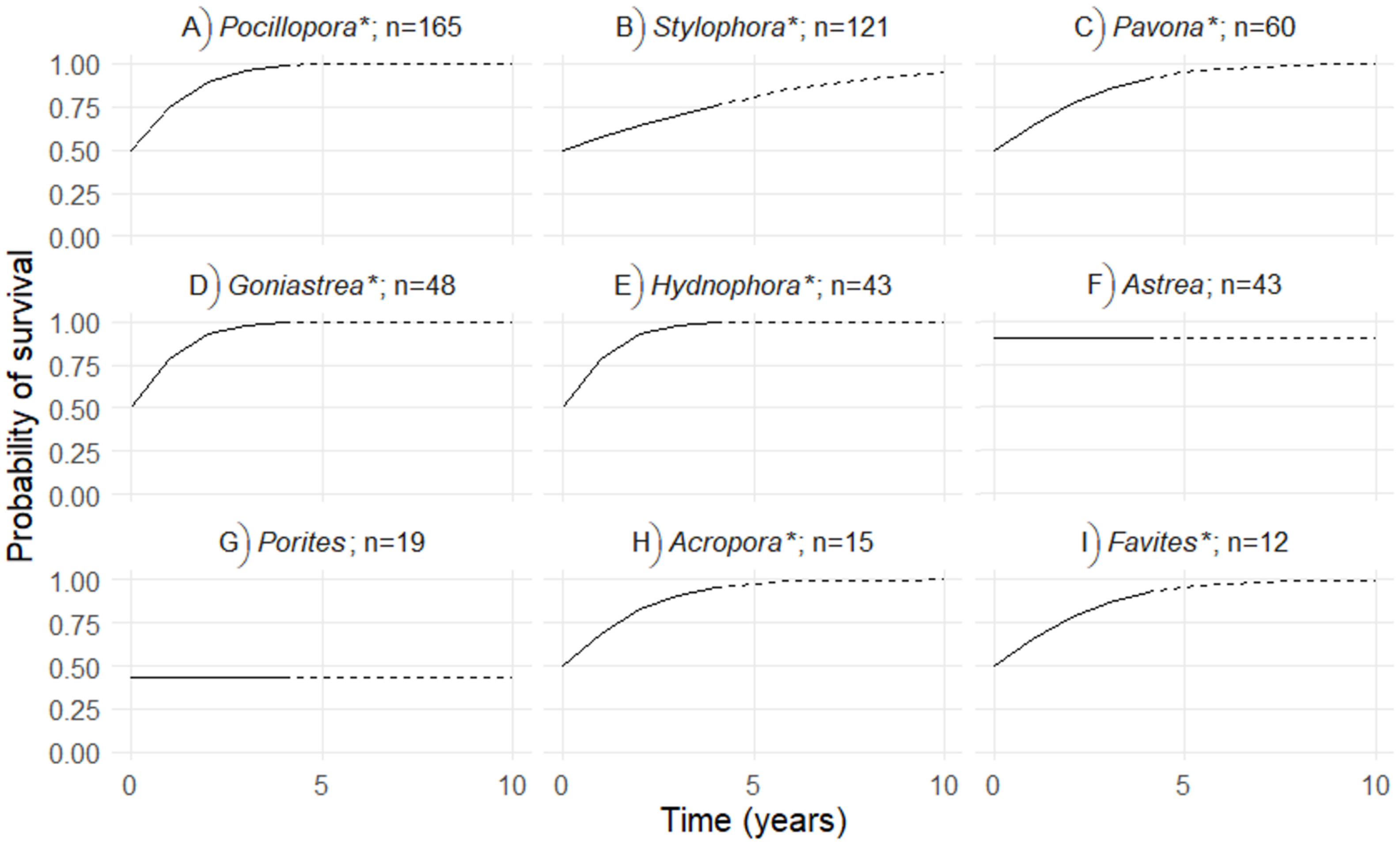


Figure S6: Estimated survival through time for coral juveniles of 9 taxa. It was computed from statistical expectations based upon best-fit parameters for models of either constant annual survival or logistic annual survival. Solid lines represent the signal fitting the data, dashed lines represent further extrapolations. Models reported are for a single habitat type, and asterisks indicate statistical support ($p<0.05$) of the logistic model relative to the constant model using a Likelihood Ratio Test. Parameters' estimate values by taxon are *Pocillopora* (s=0.80; b=1.09), *Stylophora* (s=1; b=0.29), *Pavona* (s=1; b=0.59), *Goniastrea* (s=0.95, b=1.28), *Hydnophora* (s=0.96, b=1.30), *Astrea* ($s_c$=0.90), *Porites* ($s_c$=0.43), *Acropora* (s=92, b=0.78) and *Favites* (s=1, b=0.63).
